# Mitochondrial Sequence Variation in African-American Primary Open-Angle Glaucoma Patients

**DOI:** 10.1371/journal.pone.0076627

**Published:** 2013-10-01

**Authors:** David W. Collins, Harini V. Gudiseva, Benjamin T. Trachtman, Matthew Jerrehian, Thomasine Gorry, William T. Merritt III, Allison L. Rhodes, Prithvi S. Sankar, Meredith Regina, Eydie Miller-Ellis, Joan M. O’Brien

**Affiliations:** Department of Ophthalmology, Scheie Eye Institute, University of Pennsylvania Perelman School of Medicine, Philadelphia, Pennsylvania, United States of America; Oregon Health & Science University, United States of America

## Abstract

Primary open-angle glaucoma (POAG) is a major cause of blindness and results from irreversible retinal ganglion cell damage and optic nerve degeneration. In the United States, POAG is most prevalent in African-Americans. Mitochondrial genetics and dysfunction have been implicated in POAG, and potentially pathogenic sequence variations, in particular novel transversional base substitutions, are reportedly common in mitochondrial genomes (mtDNA) from POAG patient blood. The purpose of this study was to ascertain the spectrum of sequence variation in mtDNA from African-American POAG patients and determine whether novel nonsynonymous, transversional or other potentially pathogenic sequence variations are observed more commonly in POAG cases than controls. mtDNA from African-American POAG cases (n = 22) and age-matched controls (n = 22) was analyzed by deep sequencing of a single 16,487 base pair PCR amplicon by Ion Torrent, and candidate novel variants were validated by Sanger sequencing. Sequence variants were classified and interpreted using the MITOMAP compendium of polymorphisms. 99.8% of the observed variations had been previously reported. The ratio of novel variants to POAG cases was 7-fold lower than a prior estimate. Novel mtDNA variants were present in 3 of 22 cases, novel nonsynonymous changes in 1 of 22 cases and novel transversions in 0 of 22 cases; these proportions are significantly lower (p<.0005, p<.0004, p<.0001) than estimated previously for POAG, and did not differ significantly from controls. Although it is possible that mitochondrial genetics play a role in African-Americans’ high susceptibility to POAG, it is unlikely that any mitochondrial respiratory dysfunction is due to an abnormally high incidence of novel mutations that can be detected in mtDNA from peripheral blood.

## Introduction

Primary open-angle glaucoma (POAG) is defined as a spectrum of diseases causing vision loss as a result of progressive and irreversible retinal ganglion cell damage, optic nerve degeneration and corresponding visual field loss [1]. African-Americans are disproportionately affected by POAG. A metaanalysis of 46 observational studies found that POAG prevalence in people over age 70 was 16% for blacks and 6% for whites [2], and the age-adjusted prevalence rate of POAG in African-Americans is four- to five-fold higher than whites [3].

Mitochondria are subcellular organelles descended from an ancient bacterial endosymbiont. They are responsible for energy metabolism based on oxidative phosphorylation, and play a central role in apoptosis and tissue homeostasis. Although supported and regulated by ~1,500 nuclear genes, human mitochondria possess their own genome, mtDNA, a circular molecule of 16,549 base pairs encoding 22 tRNAs, 2 ribosomal RNAs and protein components of the electron transfer chain. mtDNA variants and haplogroups have been linked to neurodegenerative diseases, including Parkinson’s disease and Alzheimer’s disease [4]. Mutations in mtDNA may result in degeneration of the optic nerve: a nonsynonymous mitochondrial mutation was first identified as the cause of Leber’s hereditary optic neuropathy (LHON) [5]. Inheritance of human mtDNA is exclusively maternal, and all humans can trace their maternal ancestry back to a single African woman, the most recent common ancestor (MRCA), who lived 194,300 +/- 32,550 years ago [6]. Sequence variants in mtDNA have accumulated in a manner proportional to divergence time, giving rise to distinct collections of ancestral variants, “haplogroups”, that reflect humanity’s African genesis and patterns of global migration. African mitochondrial haplogroups have been linked to elevated risk for POAG in Saudi patients [7].

Several lines of evidence suggest that mitochondrial genetics and function might play a role in POAG pathogenesis [8]. For example, a maternal family history of POAG is more likely than a paternal one, as would be expected from mitochondrial genetics: mothers and sisters of glaucoma patients were more likely to have glaucoma than fathers and brothers [9]. The loss of retinal ganglion cells (RGC) is characteristic of POAG, and RGC have a high energy requirement, which may make them especially vulnerable to mitochondrial dysfunction. A mitochondrial complex-I defect has been proposed as a mechanism for the loss of trabecular meshwork cells in POAG, which were found to have higher levels of reactive oxygen species and to be more sensitive to mitochondrial inhibition than normal cells; treatment with antioxidants or mitochondrial permeability transition inhibitors might inhibit disease progression [10]. Lymphocytes of POAG patients were reported to have significantly lower mean mitochondrial respiratory activity, and POAG lymphoblast cell lines were recently found to have a complex-I ATP synthesis defect [11-13]. Finally, 52% of Saudi POAG cases were found to harbor novel potentially pathogenic nonsynonymous changes in mtDNA from blood and 63% of POAG cases were found to have novel and unusual transversional (purine <-> pyrimidine) nucleotide substitutions [11]. It was hypothesized that somatic mtDNA transversions were generated in response to oxidative stress in early development or throughout life, contributing to optic nerve injury in POAG.

The mitochondrial mutations implicated in LHON have been examined in POAG patients, but do not appear to be significant risk factors, although one study of Japanese patients found 5 LHON-associated mitochondrial mutations in 551 cases, vs. none in 284 controls [14]. One investigation failed to find an association between mtDNA haplotypes and POAG [15], but included only subjects from European populations. A study of 176 Saudi POAG patients and 186 controls found that individuals with African mtDNA haplotypes were at higher risk for developing glaucoma, whereas a Eurasian mtDNA haplogroup may have a mild protective effect [7]. However, a study of Ghanaian patients concluded that mtDNA haplogroups did not confer susceptibility to POAG [16].

Mitochondrial sequence variation in POAG has not been examined in an African-American population. The objective of this study was to determine the numbers and types of previously described mtDNA polymorphisms and measure the prevalence of novel variants. A main goal was to replicate the finding that a majority of POAG patients harbor novel and potentially pathogenic variants in mtDNA, in particular transversional base substitutions [11]. This observation has been cited as evidence for mitochondrial involvement in glaucoma and could explain the systemic respiratory defects observed in cells from POAG patients, and possibly the high prevalence of POAG in African-Americans. If commonly detectable in peripheral blood, such variation could have translational utility. For example, inherited or acquired mitochondrial dysfunction in POAG may be treatable with dietary interventions [17].

## Materials and Methods

### Ethics statement

This research was conducted in accordance with the tenets of the Declaration of Helsinki under a protocol approved by the institutional review board of the University of Pennsylvania. All subjects provided informed written consent to participate in this study.

### Patient recruitment and selection

Subjects for deep mitochondrial sequencing were randomly selected from those recruited for the Primary Open-angle African-American Glaucoma Genetics (POAAGG) study within the clinical practices of the Department of Ophthalmology at the University of Pennsylvania Health System. Clinical research coordinators screened potential subjects based on institutional review board-approved inclusion/exclusion criteria. All POAAGG subjects must self-identify as Black (African-American, African descent, African Caribbean) and be age 35 or older. Subjects who met these criteria provided informed written consent, following a physician’s review of the subject’s medical record. Subjects were placed into two phenotypic categories: case or control. Case subjects were defined as those diagnosed with primary/chronic open-angle glaucoma, who had no prior diagnosis or history of ocular inflammations (e.g. uveitis), high myopia (-8 diopters or greater), other forms of glaucoma (e.g., traumatic, pseudoexfoliation, narrow/closed angle and neovascular), optic-nerve neuropathy, optic-nerve drusen and vein/artery occlusion of the retina. None of the cases had normal tension glaucoma. To ensure phenotypic rigor, POAG was defined as the presence of an open angle with both criteria employed by the LALES study [18]: "(1) congruent, characteristic or compatible glaucomatous visual ﬁeld (VF) abnormality and (2) evidence of characteristic or compatible glaucomatous optic disc damage in at least one eye. Case subjects were graded based on clinical ophthalmic examination and were required to have:

1 potential secondary causes of glaucoma excluded;
2 characteristic glaucomatous optic nerve changes in one or both eyes consisting of at least one of the following: a) excavation, neuroretinal rim thinning, notching or a nerve fiber layer defect; and b) asymmetry of the cup to disc ratio between eyes of greater than 0.2;
3 characteristic visual field defects defined by at least one of the following on two consecutive visual fields in at least one eye and consistent with optic nerve evaluation: a) glaucoma hemi field test result of outside normal limits; and b) pattern standard deviation triggered at 5% or worse."

Exclusion criteria for POAAGG controls were: a history of any form of glaucoma, vein/artery occlusion of the retina, high myopia, ocular inflammation, optic nerve neuropathy or a significant visual field loss associated with ocular disease (e.g. proliferative diabetic retinopathy). The following ocular comorbidities were documented for all subjects and did not prohibit enrollment as defined by the inclusion/exclusion criteria: macular degeneration, cataract, diabetic retinopathy, pseudophakia and posterior vitreous detachment.

A detailed ocular examination was performed on all subjects, and phenotypic data for the following variables were collected: intra-ocular pressure (at enrollment and maximum), central corneal thickness, cup to disc ratio and retinal nerve fiber layer (RNFL) thickness. Intra-ocular pressure was obtained by Goldmann applanation tonometry (GAT), and where a reliable GAT was not possible, a portable tonometer was utilized. An ultrasonic pachymeter was used to obtain central corneal thickness. The estimated cup to disc ratio was determined by the clinician’s observation of the optic nerve during a dilated fundus examination. RNFL was measured by ocular coherence tomography (OCT). The subject’s medical record was reviewed for data on additional variables: current or previous treatment with ocular medication, current or previous ocular surgery and a family history of glaucoma. All available ocular imaging reports from OCT, Heidelberg retinal tomography, visual field and fundus photography were reviewed for each subject.

787 case subjects were questioned for family history of POAG. 428 cases presented with a positive family history, and 22 of these were randomly selected for mitochondrial sequencing, along with 22 age-matched controls (+/- 5 years).

### DNA isolation and PCR amplification of mitochondrial genomes

DNA was extracted from peripheral blood using PureGene kits (Qiagen, Valencia, CA) and quantified with a Nanodrop 8000 UV spectrophotometer (Thermo Scientific, Wilmington, DE). mtDNA was enriched by polymerase chain reaction (PCR) as one long amplicon using a pair of published primers [19] and the SequalPrep Long-Range PCR kit with dNTPs (Life Technologies, Foster City, CA) in conjunction with optional enhancer B at 1x concentration. Thermal cycling was done on a BioRad iCyler (BioRad, Hercules, CA) as follows: 94 deg 2 min; 32 cycles (94 deg 10 sec, 71 deg 30 sec, 68 deg 24.5 min with increase of 20 sec per cycle); 72 deg 5 min; hold 10 deg. The presence of a single band was confirmed by gel electrophoresis and staining with ethidium bromide, and its identity was verified by bidirectional Sanger sequencing.

### Ion Torrent PGM and capillary electrophoresis Sanger sequencing

Unless otherwise noted, Ion Torrent semiconductor sequencing and capillary electrophoresis (CE) Sanger sequencing both utilized equipment, protocols and reagents supplied by Life Technologies (Foster City, CA), per manufacturer’s instructions. PCR products and sequencing libraries were purified with Agencourt AMPure XP beads (Beckman Coulter, Brea, CA). Ion Torrent sequencing libraries were constructed using Ion Xpress fragment library kits with enzymatic shearing and the Ion Xpress bar-code adapters 1-16 kit. A Pippin Prep instrument (Sage Science, Beverly, MA) was used for size selection during library construction. DNA concentrations were quantified and size distributions evaluated with an Agilent Bioanalyzer 2100 (Agilent, Santa Clara, CA). Emulsion PCR and enrichment were done with Xpress Template kit v2.0 and Ion OneTouch-ES instrument. A QuBit 2.0 fluorometer was used for quality control and to quantify Ion sphere particle enrichment. Ion sphere particles were sequenced using Ion 314 or 316 chips with 65 or 130 cycles of nucleotide incorporation on an Ion Torrent PGM instrument with Ion PGM 200 sequencing kits. The raw dataset has been deposited in the NCBI Sequence Read Archive, accession number SRA074922.

CE, Sanger sequencing was used to validate all putative novel variants identified by Ion Torrent sequencing. Additional Sanger sequencing covering >82% of the mitochondrial genome was analyzed to estimate Ion Torrent sequence accuracy and the numbers and types of false positives and false negatives, using the same PCR products used for Ion Torrent library construction. Approximately 25% of the variants inferred from Ion Torrent semiconductor sequencing were re-tested by CE, Sanger sequencing to estimate error frequencies and types. CE, Sanger sequencing utilized a published set of internal primers [20], an Applied Biosystems 3130xl genetic analyzer with 50 cm capillary array, POP-7 polymer, BigDye 3.1 cycle sequencing kits and XTerminator purification kits.

### Data analysis

Ion Torrent base calling and variant detection were performed with versions 2.0 and 2.2 of the Torrent Suite software (Life Technologies, Foster City, CA). The VariantCaller was set to “somatic” setting, with 5% as the threshold for variant reporting. To reduce the likelihood of false positives, candidate variants with mixed base calls below a filtering threshold of 25% were excluded from further analysis. Variants not confirmed by reanalysis with v2.2 of VariantCaller, by resequencing the same library a second time on Ion Torrent or by CE, Sanger sequencing were also excluded. The Integrated Genomics Viewer [21] (Broad Institute, Cambridge, MA) was used to view the alignment of individual reads. Ion Torrent and Sanger reads were assembled to the mitochondrial reference sequence NC_012920.1 (revised Cambridge Reference Sequence (rCRS)) and the complete human genome reference sequence (hg19) for analysis. Mutation nomenclature is per HGVS guidelines (www.hgvs.org) and refers to the rCRS mitochondrial reference sequence.

Sequence Analysis 5.2 software (Life Technologies, Foster City, CA) was used for CE, Sanger base calling with default settings, and chromatograms were trimmed to exclude low-quality regions (QV<25) and aligned to the rCRS with Sequencher v4.9 (GeneCodes, Ann Arbor, MI). Ion Torrent overall sequencing accuracy on mtDNA was estimated from the number of concordant calls between Sanger and Ion Torrent sequencing. False negative variants were defined as those that were detected by Sanger sequencing, but not by Ion Torrent, and false positive variants as those reported by Ion Torrent, but subsequently invalidated by Sanger. The "false negative variant rate" was defined as S/(S+B), and the "false positive variant rate" as I/(I+B), where S = variants detected by Sanger, but not Ion Torrent (false negatives), and B = variants detected by both methods (true positives) and I = variants detected by Ion Torrent, but not Sanger (false positives). S, B and I were calculated individually for transitions ((purine <-> purine: A<->G) or (pyrimidine <-> pyrimidine: C<->T)), transversions (purine <-> pyrimidine: A<->C, A<->T, G<->C, G<->T) and insertions plus deletions. Consensus sequences derived from combining the Ion Torrent and CE, Sanger results have been deposited in GenBank, accession numbers KF055290- KF055333. The MITOMAP [22] and MITOMASTER [23] resources (http://www.mitomap.org), Build 15 of the PhyloTree database (www.phylotree.org) and the mtDB database [24] were used to classify variants as novel, disease- and/or haplogroup-associated polymorphisms and for phylogenetic analysis. PolyPhen-2 [25] was used to predict the pathogenicity of nonsynonymous variants. Statistical significance using Fisher’s exact test and confidence intervals on proportions, using the modified Wald method, were computed using an online calculator (http://graphpad.com/quickcalcs) and means compared by t-test in an Excel spreadsheet (Microsoft, Redmond, WA).

## Results

### Study population

The mean age at time of enrollment was 69 years for POAG cases and 66 years for controls. 27% of POAG cases were male, vs. 36% of controls. POAG cases had significantly higher mean maximum intra-ocular pressure (IOP) and mean cup to disc ratio (Table **1**). Cupping of the optic disc is a standard measure of glaucoma progression.

**Table 1:** Selected demographic and clinical characteristics of 44 African-American POAG patients and controls (OD = right eye, OS = left eye).

	**POAG cases**		**Controls**		**Total**		**Significance**	
**Characteristic**	**N**	**Mean (SD)**	**N**	**Mean (SD)**	**Mean (SD)**	**Range**	**t-test**	**p-value**
Age at enrollment (years)	22	69 (12).	22	66 (11).	68 (11).	46-94.	0.86	0.40.
Male (%)	22	27%%	22	36%	32%	-	-^1^	0.75.
Maximum IOP OD (mm Hg)	22	26 (6.2).	22	17 (2.8).	21 (6.7).	11-41.	6.2.	<0.0001*
Maximum IOP, OS (mm Hg)	21	29 (10).	22	17 (2.7).	22 (9.2).	10-48.	5.4	<0.0001*
Cup to disc ratio OD	21	0.7 (0.2)	20	0.3 (0.1)	0.5 (0.2)	0.10-0.85	8.0	<0.0001*
Cup to disc ratio OS	19	0.7 (0.2)	20	0.3 (0.1)	0.5 (0.3)	0.10-0.95	8.1	<0.0001*

* Fisher’s exact test, two-tailed

### Validation of novel variants and estimation of error type and frequency by Sanger sequencing

All candidate novel variants identified by Ion Torrent sequencing were confirmed by CE, Sanger sequencing. Additional Sanger sequencing, primarily targeting the more variable D-loop, was done to further estimate the location, type and frequency of false positives and false negatives in the Ion Torrent data. For purposes of error estimation, 82% of the mitochondrial genome was resequenced with one or more Sanger reads, and 59,038 base pairs were screened for variants and compared to Ion Torrent results.

Most Ion Torrent errors were related to sequence context and confined to only 7 loci in mtDNA. Typically these were runs of the same base, and false negative variants, mostly insertions or deletions, were more common than false positive variants. 43 false negative errors and 6 false positive errors were found. Accordingly, Ion Torrent sequencing accuracy was estimated to be >99.9%. More than 86% of the Ion Torrent errors were false negatives involving failure to call small insertions and deletions. The "false negative variant rate" (see Methods for definition) for the Ion Torrent sequencing on mtDNA was estimated to be 0.2% for transitions, and 0% for transversions. The "false positive variant rate" was estimated to be 1.0% for transitions and 11.8% for transversions.

### Sequence variation in POAG cases and controls

**Figure 1:**
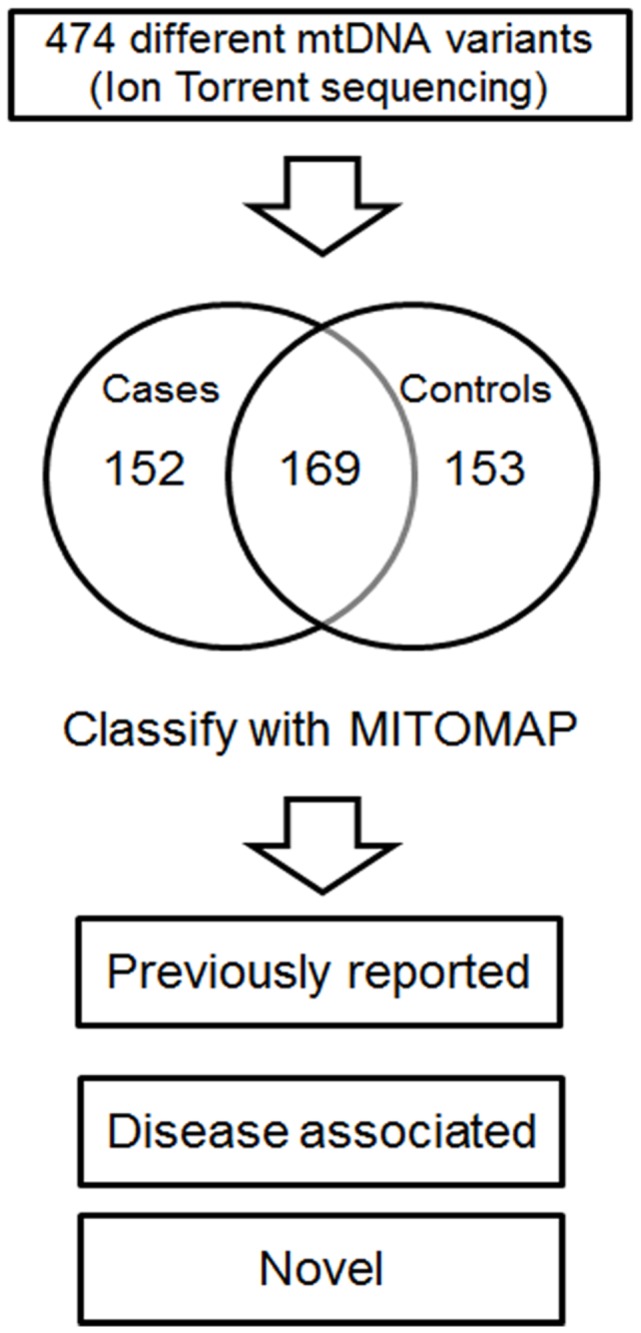
Variant identification and classification. Variations from the rCRS in African-American POAG patients and controls were identified by Ion Torrent sequencing and classified using the MITOMAP database.

Complete coverage of the single long PCR amplicon by Ion Torrent sequencing was obtained for all 44 samples. The amplicon covered 99.5% of the mitochondrial genome and was sequenced to a mean coverage depth of >1,000x. A total of 2,166 instances of variation and 474 different variants (Table **S1**) from the revised Cambridge reference sequence (rCRS) were identified and classified using MITOMAP, a compendium of polymorphisms and mutations in human mitochondrial DNA (http://www.mitomap.org/MITOMAP) (Figure **1**). Nearly all instances of variation, 99.8%, corresponded to polymorphisms that have been reported by others and are described in MITOMAP (Table **2**).

**Table 2:** Observed instances of variation from the rCRS in mtDNA from peripheral blood of 44 African-Americans.

**Classification**	**Variant type**	**POAG (n = 22)**	**Controls (n = 22)**
**Previously reported**	Transitions	1,093 (95.4%)	979 (96.0%)
	Transversions	41 (3.5%)	33 (3.2%)
	Insertions, deletions	9 (0.8%)	7 (0.7%)
**Novel variant**	Transitions	3 (0.3%)	0
	Transversions	0	1 (0.1%)
	Insertions, deletions	0	0
	Total	1,146 (100%)	1,020 (100%)

**Figure 2:**
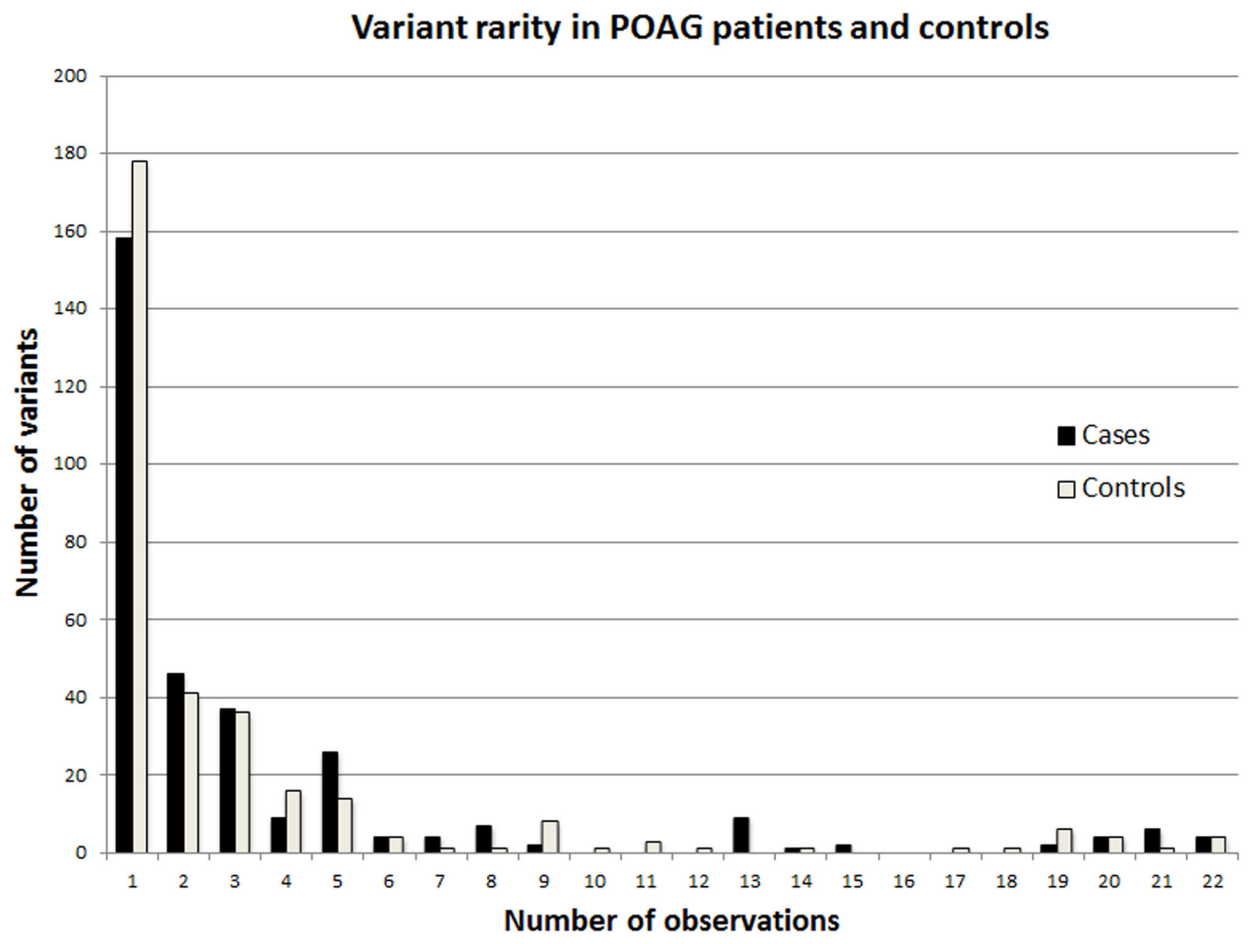
Variant rarity. The 474 different mtDNA variants, binned by number of observations in POAG cases and controls.

Of the 474 different variants, nearly identical numbers were observed to be unique to cases (152) and to controls (153), and a minority of variants (169) were observed in both cases and controls (Figure **1**). 71% of the 474 different variants were observed only once in 44 samples, i.e., most mtDNA variants were rare in the population as a whole. The number of different "rare" variants, defined here as those that were observed only once, was not greater in POAG cases than controls (Figure **2**). A few variants were observed very frequently, 17 or more times, in both cases and controls. This was expected, and these represent instances in which the rCRS itself contains a rare variant. Transitional base substitutions greatly outnumbered transversions, as expected for known human mtDNA variants. The ratio of transitions to transversions among the previously reported variants was 27:1 for POAG cases and 30:1 for controls (Table **2**).

Only four "novel" variants, defined as those not found in MITOMAP, were detected in 44 mtDNAs; three of these were found in POAG cases and one in a control (Tables **2**, **3**). Accordingly, the proportion of African-Americans with novel mtDNA variants was 9.1% (95% confidence interval: 3% to 22%), with novel variants observed in 13.6% of POAG cases (95% confidence interval: 4% to 34%), and 4.5% of controls (95% confidence interval: <0.01% to 24%). The difference in number of POAG cases (1) with novel variants from controls (3) with novel variants is not statistically significant. All three novel variants in POAG cases were transitions (Table **3**). The first, m.8274C>T, occurs in a non-coding region and has been observed in other primates (tarsier and bush baby, source: UCSC human genome browser [26] conservation track [27]) and is therefore unlikely to be pathogenic. The second, m.11813C>T, causes a Leu>Phe amino acid replacement at an imperfectly conserved residue in the ND4 gene, and was predicted to be "benign" by PolyPhen-2. The third, m.12266A>G, is located within the 5'-acceptor end of the MT-TL2 tRNA leucine gene. This A>G transition has been observed in another primate and other organisms according to the Mamit-tRNA database [28] (http://mamit-tRNA.u-strasbg.fr), so is also unlikely to be pathogenic.

**Table 3:** Variants detected in mtDNA of African-American POAG cases and controls, but not found in the MITOMAP or PhyloTree databases.

**Group**	**Variant**	**Locus**	**AA change**	**Substitution type**	**Likely pathogenic?**
**POAG**	m.8274C>T	Non-coding 7	-	Transition	No
	m.11813C>T	ND4	Leu>Phe	Transition	No
	m.12266A>G	tRNA-Leu	-	Transition	No
**Control**	m.10694A>T	ND4L	Syn.	Transversion	No

**Figure 3:**
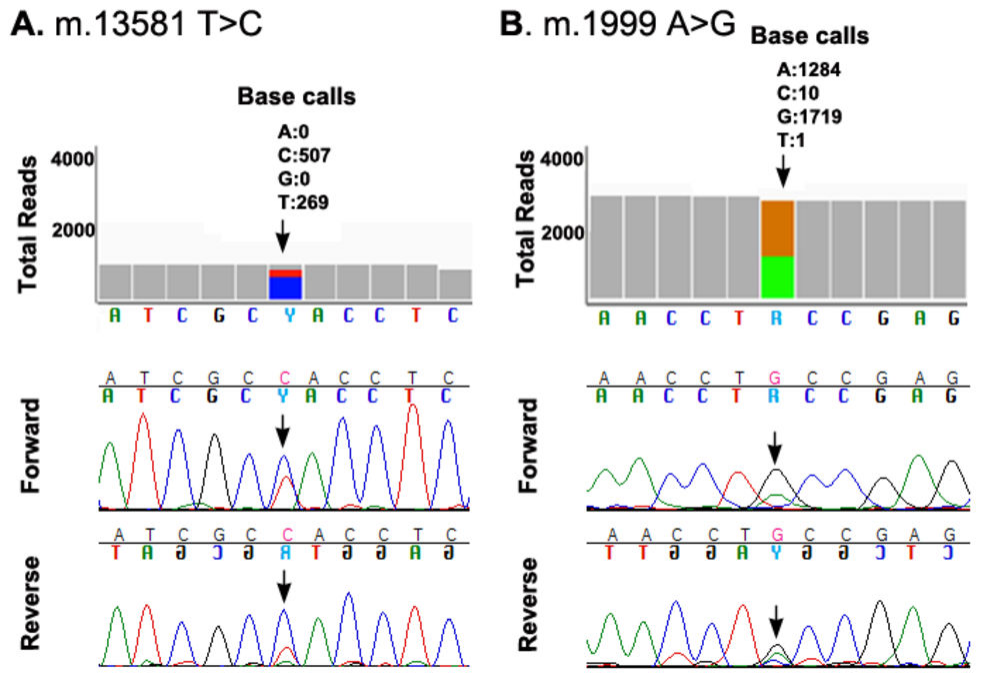
Heteroplasmic variants. Two examples of heteroplasmic variants that were detected on the basis of mixed Ion Torrent reads (top) and confirmed by CE, Sanger sequencing (bottom). One was from a POAG case (panel A) and one was from a control (panel B).

For the population as a whole (n = 44) the proportion with novel transversions was 2.3% (95% confidence interval: <0.01% to 13%). No POAG cases were found to harbor a novel transversion (purine <-> pyrimidine substitution) in mtDNA (95% confidence interval: 0% to 18%), but one instance of a novel transversion, m.10694A>T, was observed in a control (95% confidence interval: <0.01% to 24%) (Table **3**). This substitution, within the coding region of the ND4L gene, is synonymous and presumably benign. The difference between cases and controls with novel transversions was not significant (p = 1.00, Fisher’s exact test, two-tailed). No novel insertions or deletions were found (Table **2**). Instances of previously reported insertions and deletions were detected, but did not differ significantly between cases and controls (p = 0.75, Fisher’s exact test, two-tailed). Six apparently heteroplasmic variants were identified (m.310T>C, m.1999A>G, m.13581T>C, m.16150C>T, m.16390G>A, m.16399A>G), four in cases and two in controls. These were identified on the basis of mixed Ion Torrent reads, with variant frequencies ranging from 25% to 60%, and the heteroplasmy was confirmed by CE, Sanger sequencing (Figure **3**). The difference between POAG cases having heteroplasmic variants vs. controls was not statistically significant, and these variants have all been reported previously.

Polymorphisms were classified as "disease-associated" if annotated as such by MITOMAP and at least one publication has proposed a linkage to disease. A total of 171 instances of disease-associated variants of 31 different types were detected, 84 in POAG cases and 87 in controls, and 0% were transversions (Table **4**). On average, 3.8 instances of disease-associated variation were found per POAG case, vs. 4.0 per control. Variant m.16189T>C, linked to multiple diseases, was observed twice as often in controls as in cases; however this difference is not statistically significant. The differences in frequency between cases and controls for the 30 other disease-associated nucleotide changes also did not differ significantly (Fisher’s exact test, two-tailed).

**Table 4:** Disease-associated variants in POAG cases and controls.

**Variant**	**Locus**	**AA change**	**Disease association (MITOMAP)**	**POAG n = 22**	**Control n = 22**
**m.150C>T**	D-loop	-	Longevity, cervical carcinoma, HPV infection risk	9	11
**m.195T>C**	D-loop	-	Bipolar disorder-associated	13	11
**m.921T>C**	12S rRNA	-	Possibly left ventricular non-compaction-associated	0	3
**m.961T>C**	12S rRNA	-	DEAF, possibly left ventricular non-compaction-associated	0	1
**m.2352T>C**	16S rRNA	-	Possibly left ventricular non-compaction-associated	7	8
**m.3010G>A**	16S rRNA	-	Cyclic vomiting syndrome with migraine	4	1
**m.3308T>C**	ND1	Met>Thr	MELAS, DEAF enhancer, hypertension	3	3
**m.3396T>C**	ND1	Syn.	Non-syndromic hearing loss, maternally inherited DM & deafness	2	0
**m.3866T>C**	ND1	Ile>Thr	LHON (secondary mutation), limb claudication	0	1
**m.4216T>C**	ND1	Tyr>His	LHON (secondary mutation), insulin resistance	1	0
**m.5460G>A**	ND2	Ala>Thr	Alzheimer’s disease, Parkinson’s disease	1	1
**m.5655T>C**	tRNA Ala	-	DEAF enhancer	3	3
**m.6150G>A**	CO1	Val>Ile	Prostate cancer	2	1
**m.6253T>C**	CO1	Met>Thr	Prostate cancer	1	0
**m.6480G>A**	CO1	Val>Ile	Prostate cancer	1	0
**m.6663A>G**	CO1	Ile>Val	Prostate cancer	2	0
**m.8932C>T**	ATP6	Pro>Ser	Prostate cancer	2	0
**m.10398A>G**	ND3	Thr>Ala	PD protective factor, longevity, alt. cell pH, met. syn., breast cancer risk	21	19
**m.11467A>G**	ND4	Syn.	Altered brain pH	0	1
**m.12236G>A**	tRNA Ser (2)	-	DEAF	3	2
**m.12308A>G**	tRNA Leu	-	CPEO, stroke, CM, breast, renal, prostate cancer risk, alt. brain pH	0	1
**m.12372G>A**	ND5	Syn.	Altered brain pH	0	1
**m.13135G>A**	ND5	Ala>Thr	Possible hypertrophic cardiomyopathy association	1	0
**m.13708G>A**	ND5	Ala>Thr	LHON, increased MS risk, higher frequency in PD and AD	1	2
**m.14319T>C**	ND6	Asn>Asp	Parkinson’s disease, early onset	0	1
**m.15043G>A**	CYB	Syn.	Major depressive disorder	0	2
**m.15497G>A**	CYB	Gly>Ser	Obesity, exercise intolerance	0	1
**m.15812G>A**	CYB	Val>Met	LHON (secondary mutation)	0	1
**m.15927G>A**	tRNA Thr	-	Multiple sclerosis, DEAF1555 increased penetrance	0	1
**m.15942T>C**	tRNA Thr	-	Possibly left ventricular non-compaction-associated	2	1
**m.16189T>C**	D-loop	-	DM type 2, cardiomyopathy, endometrial cancer risk, mtDNA copy #, met. syn.	5	10
**Total**				84	87
**Mean observations (SD)**				3.8 (4.5)	4.0 (4.4)
**Transversions (%)**				0%	0%

### Maternal ancestry inferred from haplogroup-associated variants

**Figure 4:**
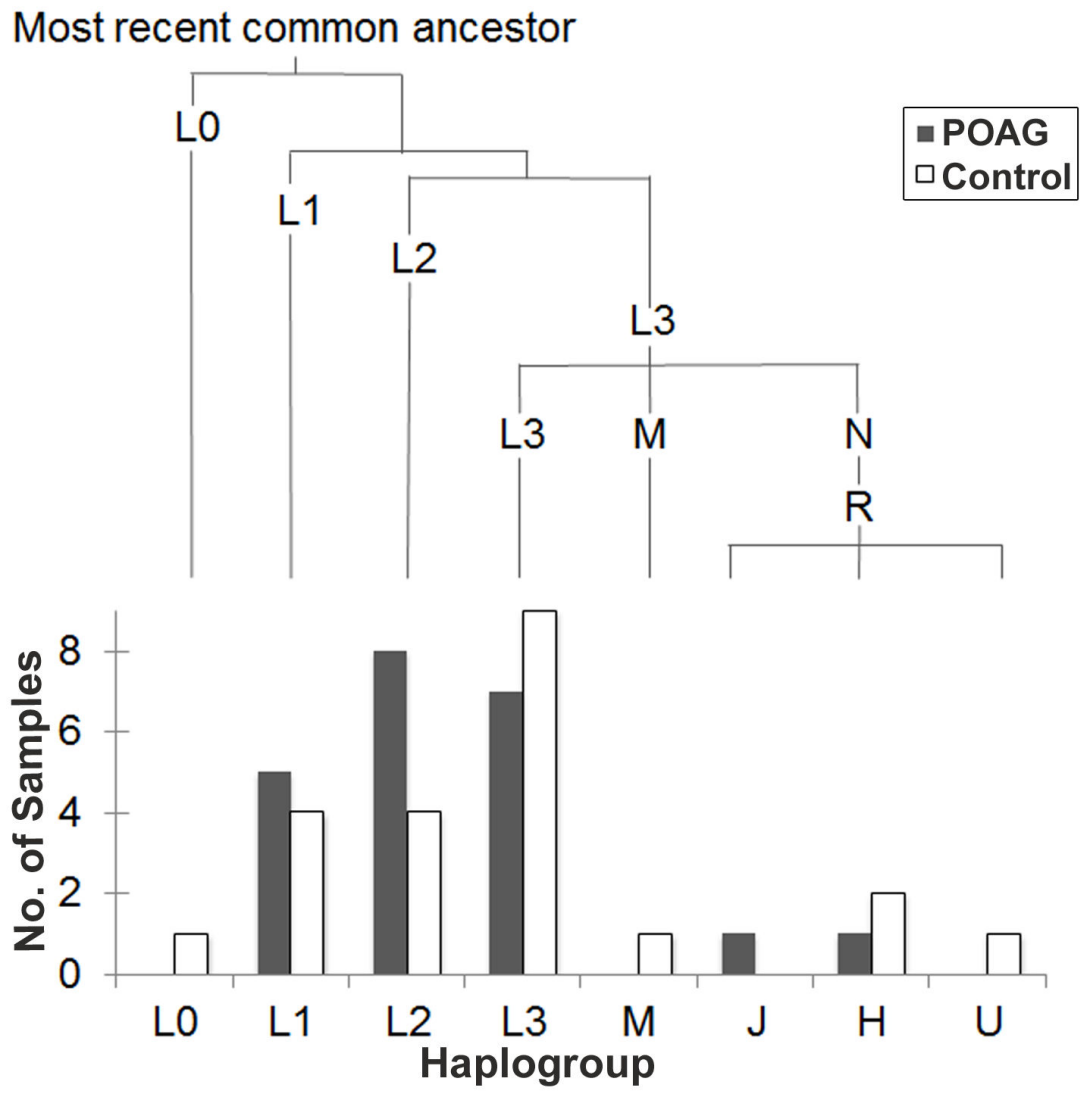
Phylogenetic relationship of 44 African-American mtDNAs, and relative numbers of POAG cases and controls, by haplogroup. Branch lengths (top) are proportional to divergence time from the most recent common ancestor, ~194,300 years before present. Sequence variation was scored relative to the rCRS, which belongs to a European (H) haplogroup.

Phylogenetic analysis with MITOMASTER placed the study participants’ mtDNA into eight major macrohaplogroups (L0, L1, L2, L3, M, J, H, U). 86% of the study population was categorized as having African mitochondrial ancestry (haplogroups L0, L1, L2, L3), 11% as having European ancestry (haplogroups J, H, U) and 2% (one person) as having Asian ancestry (haplogroup M). Twice as many cases (8) as controls (4) were found in haplogroup L2, but this difference is not significant (p = 0.31, Fisher’s exact test, two-tailed). Figure **4** depicts the phylogenetic relationship among this study’s 44 mtDNAs. African haplogroups L1, L2, and L3 predominate, accounting for 20%, 27% and 34% of the study population, respectively. The divergence times between the L0, L1, L2 and L3 African lineages are ancient relative to the European ones, which are all more recently derived from the mtDNA lineage leading to L3.

## Discussion

### Novel variants, transitions and transversions in POAG mtDNA

A 2006 study [11] reported 34 different novel mtDNA variants in 27 POAG cases, 8 of which are now present in MITOMAP, leaving 26 of these variants as currently novel. Using the same criterion, absence from MITOMAP, to define novelty, the present study found only three novel variants (Table **3**) in 22 African-American POAG cases, a proportion that is lower by seven-fold than the 2006 study and did not differ significantly from controls. The 2006 study reported novel nonsynonymous variants classified as "pathogenic" in 52% (14 of 27) POAG cases, whereas this study identified novel nonsynonymous variants in less than 5% (1 of 22) POAG cases. This difference is highly significant (p<.0004, Fisher’s exact test, two-tailed); furthermore, the sole novel nonsynonymous variant detected in an African-American POAG case (Table **3**) was not predicted to be pathogenic. Accordingly, this evidence does not support the proposition that novel nonsynonymous or pathogenic variants are found commonly in mtDNA from POAG blood.

Vertebrate mitochondrial genomes are characterized by rapid molecular evolution relative to nuclear genes and an extreme bias favoring transitional base substitutions relative to transversional substitutions. This bias varies little among the mitochondria of humans and great apes [29]. It is therefore unexpected that the 2006 study identified novel transversions in mtDNA of 17 of 27 (63%) of Saudi POAG cases, with a high ratio of transversions to transitions. The present study’s finding that 0 of 22 African-American POAG cases harbored a novel mitochondrial transversion is significantly lower (p<0.0001, Fisher’s exact test, two-tailed) and does not support the theory that novel mtDNA transversions are characteristic of POAG, or that alternative DNA damage and repair mechanisms are involved in the generation of mtDNA mutations in POAG patients.

Random sequencing errors are more likely to generate candidate mtDNA variants that appear to be: 1) *novel* (because the number of reported polymorphisms is small relative to the number of possible substitutions in mtDNA), 2) *nonsynonymous* (because most possible mtDNA substitutions correspond to open reading frames and alter coding) or 3) *transversions* (because a 2:1 ratio of transversions to transitions is expected from chance). No novel nonsynonymous transversions were detected in 44 samples. The overall concordance between Sanger and Ion Torrent sequencing was >99.9%, so is unlikely that the number of novel transversions in mtDNA of POAG cases was underestimated substantially. However, Sanger sequencing confirmed that both false negative and false positive errors were present in the Ion Torrent data. The large majority of errors were false negatives on true small insertions or deletions located at homopolymer tracts, e.g., a run of 7 C nucleotides at rCRS positions 303-309. The false negative variant rate was 0% for transversions, vs. 0.2% for transitions and the false positive variant rate was 11.8% for transversions vs. 1.0% for transitions. Accordingly, the number of transversions was more likely to be overestimated than underestimated. Another possible source of error in PCR-based mtDNA sequencing is off-target amplification of nuclear-encoded mitochondrial pseudogenes. This possibility was minimized by using long (>30 base pair) PCR primers and a single very large (16,487 base pair) amplicon for PCR enrichment. No evidence was found for significant amplification of nuclear DNA, based on absence of secondary bands on DNA gels and results from aligning Ion Torrent reads to the complete human genome reference sequence, hg19 (data not shown).

A limitation of this study was that an 82 base pair region of the D-loop, corresponding to rCRS positions 1-9 and 16,497-16,569, was outside the single PCR amplicon, i.e., 0.5% of each genome was not amplified, and any variants in this region were not detected. Candidate variants called with frequencies <25% were excluded from the analysis to reduce the likelihood of false positives, so if true low-level heteroplasmic variants were present, these are likely to have been missed as well. We did not attempt to characterize the Ion Torrent PGM’s sensitivity to low-level mitochondrial heteroplasmy; however, our results suggest it is likely to be position-specific and dependent on factors such as read depth, sequence context and type of genetic alteration. Consistent with a recent study [30], we found the Ion Torrent’s error rate on insertions and deletions was much higher than for substitutions. The practice of equating variant "novelty" with non-registration in MITOMAP has been criticized on the basis that other approaches, such as Google searching, may be more sensitive to identifying previously observed variants [31]. Accordingly, using a more stringent criterion to define "novelty" may result in estimates for the proportion of POAG patients and controls harboring a novel variant that are even lower than the 9.1% reported here.

### Implications for POAG pathogenesis in African-Americans

The functional significance, if any, of the individual novel, previously reported and disease-associated variants for POAG for African-Americans was outside the scope of this study, but deserves further investigation. This study lacked the statistical power needed to detect effects of individual sequence variants; the large majority of variants were rare, only observed once in cases or controls. The 31 disease-associated variants (Table **4**) are a subset of polymorphisms for which a linkage to a disease has been proposed in one or more publications. However, distinguishing mildly pathogenic mitochondrial mutations from benign variants is challenging, with interpretation subject to controversy. A particular mtDNA variant may be both deleterious and beneficial depending on its haplogroup and environmental context [32]. For example, a European haplogroup J background has been shown to increase the penetrance of primary LHON mutations and to sensitize cells to environmental insults, such as exposure to hexane [33].

The great diversity in mtDNA ancestral haplogroups present in African-Americans adds further complexity to interpreting sequence variation. Although this study only examined 44 people, the deepest roots of human ancestry are represented (Figure **4**). At least 20% of the study population was assigned to each of the African haplogroups L1, L2 and L3. These three are estimated to have diverged approximately 142,300 +/-38,200 years ago [6]. Accordingly, future studies of mitochondrial variation in African-Americans and its possible relationship to POAG might be stratified to ensure that the major haplogroups have balanced representation in cases and controls, so as not to confound ancient and recent variation.

A larger total number of variants was observed in cases (1,146) than in controls (1,020) (Table **2**). This likely reflects differences in the ancestral composition of the two groups, in conjuction with the use of the rCRS, a European haplogroup H mtDNA, as the reference sequence. If, for example, the African-American POAG patients had been compared to a hypothetical control group with exclusively European maternal ancestry, one would expect to observe fewer variants in that control group, on account of its recent common ancestry with the rCRS. The control group for this study was indeed found to contain fewer L1 and L2 mtDNAs, which are distantly related to the rCRS (Figure **4**). So the direction of systematic bias from imperfectly balanced ancestry plus use of the European rCRS to identify "variants" was to favor variant identification in the POAG group over controls. Such a bias might be controlled for in future studies by scoring variation relative to a derived common ancestral sequence, such as the Reconstructed Sapiens Reference Sequence [34], which incoporates recent data from Neanderthal mtDNAs.

## Conclusions

African-American POAG cases closely resembled controls with respect to frequency and types of sequence variation in mtDNA, with very similar numbers of novel variants, heteroplasmic variants, transitions, transversions, insertions, deletions and disease-associated polymorphisms. This work did not directly address the issue of whether mitochondrial dysfunction may contribute to POAG pathogenesis. However it does suggest that if mitochondrial functional defects are found in African-American POAG patients, as in other populations, these defects are unlikely to be explained or diagnosed by an unusual mutational spectrum in mtDNA that is detectable in peripheral blood. Future studies will require larger cohorts and could consider ancestral haplogroups, somatic mtDNA variation within the eye, variation in nuclear mitochondrial genes and environmental exposures that may impair mitochondrial function.

## Supporting Information

Table S1 **Complete list of mtDNA variants inferred from Ion Torrent sequencing and numbers of observations.**
(XLS)
